# COVID-19 vaccine hesitancy among different population groups in China: a national multicenter online survey

**DOI:** 10.1186/s12879-022-07111-0

**Published:** 2022-02-14

**Authors:** Yiman Huang, Xiaoyou Su, Weijun Xiao, Hao Wang, Mingyu Si, Wenjun Wang, Xiaofen Gu, Li Ma, Li Li, Shaokai Zhang, Chunxia Yang, Yanqin Yu, Youlin Qiao

**Affiliations:** 1 School of Population Medicine and Public Health, Chinese Academy of Medical Sciences and Peking Union Medical College, Beijing, China; 2 School of Nursing, Jining Medical University, Jining, Shandong China; 3 Affiliated Tumor Hospital, Xinjiang Medical University, Urumqi, China; 4 Public Health School, Dalian Medical University, Dalian, China; 5 Department of Clinical Research, The First Affiliated Hospital, Jinan University, Guangzhou, China; 6 Henan Cancer Hospital, Affiliate Cancer Hospital of Zhengzhou University, Zhengzhou, China; 7 Department of Epidemiology and Biostatistics, West China School of Public Health and West China Fourth Hospital, Sichuan University, Chengdu, Sichuan China; 8 School of Public Health, Department of Clinical Research, Baotou Medical College, Baotou, China; 9 Department of Cancer Epidemiology, National Cancer Center/National Clinical Research Center for Cancer/Cancer Hospital, Chinese Academy of Medical Sciences and Peking Union Medical College, 17 South Panjiayuan, Chaoyang District, Beijing, China

**Keywords:** Vaccine hesitancy, COVID-19, Infodemic, Medical workers, China

## Abstract

**Background:**

COVID-19 vaccine has been available in China since the beginning of the 2021, however, certain numbers of people are reluctant for some reasons to vaccinate. The high vaccine coverage is crucial for controlling disease transmission, however, the vaccine hesitancy might be a barrier to the establishment of sufficient herd immunization. This study aims to investigate the prevalence of the COVID-19 vaccine hesitancy among different population groups, and explore common barriers and facilitators to vaccination decisions.

**Methods:**

The current survey was performed among Chinese students, public health professionals, medical workers and general population from January to March 2021 from seven cities in China. The questionnaire contained sociodemographic information, concerns about infection with COVID-19, general vaccination behaviors and attitudes, the General Vaccine Hesitancy Scale, the COVID-19 Vaccine Hesitancy Scale and other potential factors. Univariate analysis was conducted by chi-squared test, and variables significant at *P* < 0.10 were then included in a multivariable regression model.

**Results:**

The prevalence of COVID-19 vaccine hesitancy was 15.6% in our study, and 23.9% of students, 21.2% of the general population, 13.1% of medical workers, and 10.4% of public health professionals had vaccine hesitancy. The results of multivariate analysis indicated that participants who had received negative information of COVID-19 vaccine (OR: 1.563, 95% CI: 1.229–1.986) and who had doubts about the information source (OR: 2.157, 95% CI: 1.697–2.742) were more likely to have vaccine hesitancy. While those who needed transparent information about COVID-19 vaccine (OR: 0.722, 95% CI: 0.535–0.973) and who would get COVID-19 vaccine if doctors recommended (OR: 0.176, 95% CI: 0.132–0.234) were less likely to have COVID-19 vaccine hesitancy.

**Conclusions:**

Given recommendations from medical workers about vaccination can motivate people to accept COVID-19 vaccination, appropriate training in knowledge about vaccines and communication skills are necessary for them to increase public’s willingness of vaccination. Reducing the spread of misinformation and disseminating facts in a timely and accurate way will likely reduce vaccine hesitancy. Moreover, to establish suitable communication strategies and information exchange platforms between the government and the public and a warning system on infodemic would be helpful to improve public’s confidence in vaccination.

**Supplementary Information:**

The online version contains supplementary material available at 10.1186/s12879-022-07111-0.

## Background

COVID-19 (coronavirus disease 2019) was first noticed in late 2019 and is still ravaging the world currently [1]. It caused an unprecedented crisis for global public health and enormous disease burden while severely disrupting societies and economies. Since actions such as compulsory mask wearing, lockdowns and social distancing have exhausted the world from the beginning of the pandemic, effective COVID-19 vaccine is the most powerful way to curb the disease [2, 3]. Vaccination was recognized as one of the most successful public health measures, and have contributed to the decline in mortality and morbidity of various infectious diseases [4]. Herd immunization with vaccination may protect the general public against COVID-19 infection and hence stop generating large outbreak. However, certain number of people are still reluctant to receive the vaccination due to vaccine hesitancy, which leads to a relatively low vaccination coverage [5].

Vaccine hesitancy refers to delay in acceptance or refusal of vaccination despite availability of vaccination services and is complex and context specific, varying across time, place, and vaccines [6]. Vaccine hesitancy can result in apparently increases in outbreaks, morbidity and mortality of some vaccine-preventable diseases. Indeed, the concept of “vaccine hesitancy” has been considered by the World Health Organization (WHO) as “one of the top-ten threats to global health” [7]. Previous studies reported that 88.6% respondents from China would take a “proven, safe and effective vaccine”, 76.63% of Chinese healthcare workers would accept the vaccine, and approximately 68% of participants in the United States were supportive of being vaccinated for COVID-19 [8–12]. Moreover, an online survey found that about one-third of the participants in Turkey and 14% in the UK were unsure about getting COVID-19 vaccine [13]. In general, some people have hesitated or will hesitate to get COVID-19 vaccine for certain reasons, these reasons may be related to their own experience, knowledge of vaccines, and previous immunization behavior, including low perceived infectious risk, worry about possible side effects, “wait and see” attitude, concerns about safety and efficacy, and concerns over the short period of time of vaccine development, etc. [14–21].

China started rolling out COVID-19 vaccines in late 2020, prioritizing healthcare and other front-line workers and those with high-risk health conditions. Subsequently, vaccination in China was available to people aged 18 years and older and then to those aged 3–11 years. As of 8 December 2021, approximately 2575 million vaccine doses have been administered [22]. However, a portion of the public still have COVID-19 vaccine hesitancy and the prevalence of hesitancy among different populations is not clear. It is of importance to investigate the prevalence of public’s hesitancy on COVID-19 vaccines and related factors in China, and address the needs in implementing evidence-based interventions to increase the vaccination rate.

We hypothesized that there were people in China who were hesitant about the COVID-19 vaccine, and the prevalence of COVID-19 vaccine hesitancy among different population groups varied. Therefore, the present study aims to: (1) investigate the prevalence of vaccine hesitancy towards the COVID-19 vaccine among different population groups from seven geographical territories of China; (2) explore the characteristics of different groups about vaccine hesitancy and their common barriers and facilitators to vaccination decisions.

## Methods

### Study design and participants

The current study was performed from January to March 2021 among Chinese from seven cities (from Henan Province, Sichuan Province, Shandong Province, Guangdong Province, Inner Mongolia, Xinjiang Uygur Autonomous Region, and Liaoning Province respectively) located in seven geographical territories of China by distributing an online structural questionnaire via an investigation platform named Wenjuanxing. The participants were recruited from four different population groups, including students, public health professionals, medical workers and general population.

The sample size was calculated using a margin of error of 5%, a confidence level of 95%, a response rate of 50% and a previous estimate rate of vaccine hesitancy of 32.2%, giving a minimum sample size of 671 [23, 24]. The snowball sampling was used to recruit the potential study participants. We initially invited investigators from the seven cooperative institutions, and they distributed the questionnaire to the people meeting the inclusion criteria.

The eligibility criteria included age more than or equal to 18 and an ability to read, understand and complete an online questionnaire. Those who were younger than 18, had barriers to use mobile phone or computer, or had cognitive impairment were excluded. The medical workers were recruited from hospital departments such as respiratory and critical care medicine, general surgery, and nephrology department, while hospital administrators were excluded from medical workers group in our study. The public health professionals were recruited from local CDCs in China, most from the communicable disease control and prevention department, immunization program department and preventive health department. The student group was recruited from students enrolled in universities. To confirm the quality of the online survey, the members of research team were trained on data collection and inclusion criteria procedure.

### Measurements

The survey questionnaire contained sociodemographic information, concerns about infection with COVID-19, general vaccination behavior and attitudes, the General Vaccine Hesitancy Scale, the COVID-19 Vaccine Hesitancy Scale and other potential factors of vaccine hesitancy.

#### Sociodemographic information

Sociodemographic variables included age, gender, ethnicity, residence place, marital status, education level, household income (during past 1 year), smoking and drinking history, and COVID-19 test results.

#### Concerns about infection with COVID-19

We assessed the concerns of participants about infection with COVID-19 by a 3-items scale, including “I am scared about getting infected with COVID-19” “The possibility of getting infected with COVID-19 in the future concerns me” and “I don’t really worry about getting infected with COVID-19”. This 3-item scale was developed and utilized from several studies, participants responded to each item on a 5-point Likert-type scale (1 = strongly disagree to 5 = strongly agree) [25, 26]. After reverse coded the last item, the three items were highly correlated with satisfactory reliability (Cronbach’s α = 0.702).

#### The General Vaccine Hesitancy Scale

The General Vaccine Hesitancy Scale composed of 10 items which was revised on the base of previous studies [27, 28]. In this study, we used the 10 items of the Vaccine Hesitancy Scale (VHS) developed by the SAGE Working Group on Vaccine Hesitancy that are measured on a five-point Likert-type rating scale ranging from “strongly disagree” to “strongly agree”. Corresponding changes were made to the wordings of the 10 items to make the study participants fully understand the meanings. We reversed seven items in the scoring of the scale so that higher scores indicated more hesitancy on all items. The reliability of the General Vaccine Hesitancy Scale was satisfactory in our study (Cronbach’s α = 0.930).

#### The COVID-19 Vaccine Hesitancy Scale

The COVID-19 Vaccine Hesitancy Scale was measured by 15 items based on previous studies to identify vaccine-hesitant parents [29, 30]. We collapsed responses of scale items into 3 categories: hesitant responses, “not sure or don’t know”, and non-hesitant responses. The specific items and scoring rules of this scale can be found in Additional file 1: Table S1. The raw total score was calculated by summing each item’s score, ranging from 0 to 30. We used simple linear transformation to convert this raw score to a 0–100 scale, the score was higher than or equal to 50 was indicated having COVID-19 vaccine hesitancy [30]. The Cronbach’s alpha of the 15-item scale in the current study was 0.755.

#### General vaccination behavior and attitudes

The questionnaire used a series of questions to investigate respondents’ vaccination history, including the following items: “Do you agree that vaccines can protect you from diseases?” “Do you agree that you will get all vaccines that National Immunization Program or government recommended?” “Have you ever hesitated to get vaccination?” “Have you ever refused to get vaccination?” “Have you ever hesitated or refused to be get Pneumococcal Vaccine?” “Have you ever hesitated or refused to get Influenza Vaccine?”.

#### Potential factors of COVID-19 vaccine hesitancy

In this section, the following items were designed to explore the barriers of getting COVID-19 vaccine and related factors of COVID-19 vaccine hesitancy: (1) individual attitudes to COVID-19: “Do you agree that COVID-19 epidemic is a severe problem affecting the health of the community?” “Do you agree that COVID-19 will be a great threat to your health if you are infected?” (2) significant people’s advice: “Do you agree that you will get COVID-19 vaccine if doctors recommend?”, “Do you agree that the advice of your family members or friends will affect your intention of getting COVID-19 vaccine?” (3) information about COVID-19 vaccine: “Do you need transparent information about COVID-19 vaccine development, efficacy and safety?” “Do you have doubts about the source of information about the COVID-19 vaccine?” “Have you ever received negative information about getting the COVID-19 vaccine?” “Would you like to get the COVID-19 vaccine after receiving the negative information about it?” (4) cost or time to get COVID-19 vaccine: “Do you agree that the time costs in waiting for the vaccination or staying at the clinic will be a barrier for you to get COVID-19 vaccine?” “Do you agree that the environment of the clinic will be a barrier for you to get COVID-19 vaccine?” “Do you agree that the cost of going to the clinic will be a barrier for you to get COVID-19 vaccine?” (5) personal conditions: “Do you agree that you have no need of getting COVID-19 vaccine because you are healthy?” “Have you gotten emergency COVID-19 vaccination for some reasons?”.

### Statistical analysis

This study described the sociodemographic information by counts and proportions among participants. The chi-squared test was utilized to compared the differences of various factors in different population groups. Survey responses were combined into two categories (having or not having COVID-19 vaccine hesitancy) according to whether the score of the COVID-19 Vaccine Hesitancy Scale was higher than or equal to 50. And ordinal regression models were ran to examine demographic and attitudinal factors predictive of respondents' hesitancy to get vaccinated against COVID-19. To identify suitable candidate variables for regression models, univariate analysis was first conducted by chi-squared test, and variables were significant at P < 0.10 were then included in a multivariable regression model. The level of significance was set at P < 0.05. Data were analyzed by using SPSS version 24.0.

## Results

### Sociodemographic characteristics

Table 1 summarized the characteristics distributions of participants by different population groups. A total of 4289 respondents (response rate 95.37%) completed the online questionnaire, and 62 questionnaires were excluded due to the age limitations. Of them, there were 2656 (62.8%) medical workers, 753 (17.8%) students, 434 (10.3%) general population, and 384 (9.1%) public health professionals. The mean age was 33.02 years old. In total, 2818 (66.7%) respondents were female, 89.1% of them were Han ethnicity, the majority of participants (85.6%) lived in urban areas and 56.9% of them were married, 41.3% were single. The education level of most of them (93.8%) were college and above. For the household income during the past year, nearly half of them were in 50,000–100,000 Yuan per year. Most of participants self-reported they have not ever smoked (88.3%) or drank (84.6%).

**Table 1:** Sociodemographic characteristics of participants by different population groups

Variables	Medical workers (n = 2656)	Students (n = 753)	General population (n = 434)	Public health professionals (n = 384)	Total (n = 4227)
Age (years) ($mean\pm SD)$	35.89 $\pm$ 9.33	22.47 $\pm$ 3.13	29.73 $\pm$ 7.78	37.52 $\pm$ 9.03	33.02 $\pm$ 9.90
Gender, n (%)					
Male	732 (27.6)	334 (44.4)	193 (44.5)	150 (39.1)	1409 (33.3)
Female	1924 (72.4)	419 (55.6)	241 (55.5)	234 (60.9)	2818 (66.7)
Ethnicity, n (%)					
Han	2339 (88.1)	654 (86.9)	415 (95.6)	357 (93.0)	3765 (89.1)
Other	317 (11.9)	99 (13.1)	19 (4.4)	27 (7.0)	462 (10.9)
Residence place, n (%)					
Urban	2408 (90.7)	496 (65.9)	355 (81.8)	361 (94.0)	3620 (85.6)
Rural	248 (9.3)	257(34.1)	79 (18.2)	23 (6.0)	607 (14.4)
Marital status, n (%)					
Single	667 (25.1)	721 (95.8)	263 (60.6)	93 (24.2)	1744 (41.3)
Married	1927 (72.6)	27 (3.6)	165 (38.0)	285 (74.2)	2404 (56.9)
Others	62 (2.3)	5 (0.7)	6 (1.4)	6 (1.6)	79 (1.9)
Education level, n (%)					
≤ High school	160 (6.0)	25 (3.3)	53 (12.2)	22 (5.7)	260 (6.2)
College and above	2496 (94.0)	728 (96.7)	381 (87.8)	362 (94.3)	3967 (93.8)
Household income (past 1 year), n (%)					
≤ 40,000 Yuan	431 (16.2)	256 (34.0)	84 (19.4)	43 (11.2)	814 (19.3)
50,000–100,000 Yuan	1233 (46.4)	285 (37.8)	185 (42.6)	132 (34.4)	1835 (43.4)
110,000–350,000 Yuan	920 (34.6)	178 (23.6)	137 (31.6)	193 (50.3)	1428 (33.8)
> 350,000 Yuan	72 (2.7)	34 (4.5)	28 (6.5)	16 (4.2)	150 (3.5)
Ever smoking, n (%)					
No	2369 (89.2)	679 (90.2)	347 (80.0)	339 (88.3)	3734 (88.3)
Yes	287 (10.8)	74 (9.8)	87 (20.0)	45 (11.7)	493 (11.7)
Ever drinking, n (%)					
No	2276 (85.7)	668 (88.7)	329 (75.8)	303 (78.9)	3576 (84.6)
Yes	380 (14.3)	85 (11.3)	105 (24.2)	81 (21.1)	651 (15.4)
COVID-19 test results, n (%)					
Positive	12 (0.5)	19 (2.5)	6 (1.4)	2 (0.5)	39 (0.9)
Negative	2056 (77.4)	438 (58.2)	272 (62.7)	332 (86.5)	3098 (73.3)
Haven’t tested	588 (22.1)	296 (39.3)	156 (35.9)	50 (13.0)	1090 (25.8)

### Comparison of general vaccination behavior and vaccine hesitancy among different population groups

Differences of general vaccination behavior and vaccine hesitancy in different population groups were shown in Table 2. For public health professionals, 94.3% of them agreed that vaccines can protect them from diseases, which was higher than that in medical workers (90.5%), students (87.5%) and general population (88.7%). Among medical workers, the majority of them (80.0%) agreed that they will get all vaccines that the National Immunization Program or government recommended, however, a lower proportion was in students (76.5%), general population (72.6%) and public health professionals (75.0%). When the participants were asked about whether they were hesitated to get vaccination, 39.4% of general population were hesitant and it was higher than in other three population groups. Regarding the sorts of vaccines they hesitated or refused, general population ranked the highest on hesitation in receiving pneumococcal vaccine (27.4%) and health workers ranked the highest in refusing pneumococcal vaccine (9.8%). In Comparation of the General Vaccine Hesitancy Scale scores in different groups, medical workers had a higher score (20.33 $\pm$ 8.59) than students (20.32 $\pm$ 8.11), general population (19.87 $\pm$ 7.61) and public health professionals (18.48 $\pm$ 7.51).

**Table 2:** Comparison of general vaccination behavior and vaccine hesitancy among different population groups

Variables	Medical workers	Students	General population	Public health professionals	*P*
	n (%)	n (%)	n (%)	n (%)	
Do you agree that vaccines can protect you from diseases?					
No	253 (9.5)	94 (12.5)	49 (11.3)	22 (5.7)	0.002
Yes	2403 (90.5)	659 (87.5)	385 (88.7)	362 (94.3)	
Do you agree that you will get all vaccines that the National Immunization Program or government recommended?					
No	530 (20.0)	177 (23.5)	119 (27.4)	96 (25.0)	0.001
Yes	2126 (80.0)	576 (76.5)	315 (72.6)	288 (75.0)	
Have you ever hesitated to get vaccination?					
No	1806 (68.0)	506 (67.2)	263 (60.6)	257 (66.9)	0.026
Yes	850 (32.0)	247 (32.8)	171 (39.4)	127 (33.1)	
Have you ever refused to get vaccination?					
No	2222 (83.7)	646 (85.8)	365 (84.1)	320 (83.3)	0.542
Yes	434 (16.3)	107 (14.2)	69 (15.9)	64 (16.7)	
Have you ever hesitated or refused to get Pneumococcal Vaccine?					
No	1759 (66.2)	509 (67.6)	293 (67.5)	267 (69.5)	< 0.001
Ever hesitated	636 (23.9)	204 (27.1)	119 (27.4)	82 (21.4)	
Ever refused	261 (9.8)	40 (5.3)	22 (5.1)	35 (9.1)	
Have you ever hesitated or refused to get Influenza Vaccine?					
No	1789 (67.4)	514 (68.3)	294 (67.7)	274 (71.4)	0.180
Ever hesitated	622 (23.4)	188 (25.0)	109 (25.1)	84 (21.9)	
Ever refused	245 (9.2)	51 (6.8)	31 (7.1)	26 (6.8)	
The General Vaccine Hesitancy Scale scores					
$m\pm SD$	20.33 $\pm$ 8.59	20.32 $\pm$ 8.11	19.87 $\pm$ 7.61	18.48 $\pm$ 7.51	0.001

### Comparison of COVID-19 vaccine hesitancy and potential factors among different population groups

In this study, 15.6% (661) of participants were observed having COVID-19 vaccine hesitancy. Among different groups, 180 (23.9%) student, 92 (21.2%) general population, 349 (13.1%) medical workers and 40 (10.4%) public health professionals self-reported having COVID-19 vaccine hesitancy. Compared to medical workers, students and public health professionals, the general population had the higher score (10.06 $\pm$ 1.79) of the concerns about infection with COVID-19. Regarding to the attitudes of respondents to COVID-19 epidemic, 75.8% of public health professionals agreed that COVID-19 epidemic is a severe problem affecting people’s health, which was significantly higher than other groups. And a statistically higher proportion of medical workers (89.9%) agreed that COVID-19 will be a great threat to their health if they infected compared to other 3 groups.

Among different groups, 94.3% of public health professionals would get COVID-19 vaccine if doctors recommend, 57.6% of students’ vaccination intention would be affected by their family members or friends, the ratings were significantly higher. Regarding the responses of the information about COVID-19 vaccine, 90.1% of public health professionals reported they need transparent information about COVID-19 vaccine and 63.5% had received negative information, 42.0% of students had doubts about the source of information. When participants were asked if the time costs in waiting for the vaccination or staying at the clinic would be barriers for them to vaccinate, a higher proportion of students were responded “Yes” compared to other three groups. For public health professionals, 9.6% of them self-reported that they have no need of getting COVID-19 vaccine because they are healthy, however, 42.2% of them had gotten emergency COVID-19 vaccination for some reasons (Table 3).

**Table 3:** Comparison of COVID-19 vaccine hesitancy and potential factors among different population groups

Variables	Medical workers	Students	General population	Public health professionals	*P*
	n (%)	n (%)	n (%)	n (%)	
Have COVID-19 vaccine hesitancy					
No	2307 (86.9)	573 (76.1)	342 (78.8)	344 (89.6)	< 0.001
Yes	349 (13.1)	180 (23.9)	92 (21.2)	40 (10.4)	
Concerns about infection with COVID-19					
$m\pm SD$	9.83 $\pm$ 2.10	9.80 $\pm$ 2.13	10.06 $\pm$ 1.79	9.56 $\pm$ 1.88	0.007
Do you agree that COVID-19 epidemic is a severe problem affecting the health of the community?					
No	698 (26.3)	242 (32.1)	146 (33.6)	93 (24.2)	< 0.001
Yes	1958 (73.7)	511 (67.9)	288 (66.4)	291 (75.8)	
Do you agree that COVID-19 will be a great threat to your health if you are infected?					
No	269 (10.1)	108 (14.3)	47 (10.8)	46 (12.0)	0.012
Yes	2387 (89.9)	645 (85.7)	387 (89.2)	338 (88.0)	
Do you agree that you will get COVID-19 vaccine if doctors recommend?					
No	221 (8.3)	101 (13.4)	68 (15.7)	22 (5.7)	< 0.001
Yes	2435 (91.7)	652 (86.6)	366 (84.3)	2435 (94.3)	
Do you agree that the advice of your family members or friends will affect your intention of getting COVID-19 vaccine?					
No	1721 (64.8)	319 (42.4)	237 (54.6)	254 (66.1)	< 0.001
Yes	935 (35.2)	434 (57.6)	197 (45.4)	130 (33.9)	
Do you need transparent information about COVID-19 vaccine development, efficacy and safety?					
No	456 (17.2)	142 (18.9)	62 (14.3)	38 (9.9)	0.001
Yes	2200 (82.8)	611 (81.1)	372 (85.7)	346 (90.1)	
Do you have doubts about the source of information about the COVID-19 vaccine?					
No	2064 (77.7)	437 (58.0)	276 (63.6)	308 (80.2)	< 0.001
Yes	592 (22.3)	316 (42.0)	158 (36.4)	76 (19.8)	
Have you ever received negative information about getting the COVID-19 vaccine?					
No	1260 (47.4)	373 (49.5)	203 (46.8)	140 (36.5)	< 0.001
Yes	1396 (52.6)	380 (50.5)	231 (53.2)	244 (63.5)	
Would you like to get the COVID-19 vaccine after receiving the negative information about it?					
No	932 (35.1)	357 (47.4)	205 (47.2)	104 (27.1)	< 0.001
Yes	1724 (64.9)	396 (52.6)	229 (52.8)	280 (72.9)	
Do you agree that the time costs in waiting for the vaccination or staying at the clinic will be a barrier for you to get COVID-19 vaccine?					
No	2033 (76.5)	444 (59.0)	290 (66.8)	281 (73.2)	< 0.001
Yes	623 (23.5)	309 (41.0)	144 (33.2)	103 (26.8)	
Do you agree that the environment of the clinic will be a barrier for you to get COVID-19 vaccine?					
No	1708 (64.3)	331 (44.0)	208 (47.9)	217 (56.5)	< 0.001
Yes	948 (35.7)	422 (56.0)	226 (52.1)	167 (43.5)	
Do you agree that the costs of going to the clinic will be a barrier for you to get COVID-19 vaccine?					
No	2011 (75.7)	376 (49.9)	271 (62.4)	270 (70.3)	< 0.001
Yes	645 (24.3)	377 (50.1)	163 (37.6)	114 (29.7)	
Do you agree that you have no need of getting COVID-19 vaccine because you are healthy?					
No	2364 (89.0)	582 (77.3)	354 (81.6)	347 (90.4)	< 0.001
Yes	292 (11.0)	171 (22.7)	80 (18.4)	37 (9.6)	
Have you gotten emergency COVID-19 vaccination for some reasons?					
No	1610 (60.6)	638 (84.7)	373 (85.9)	222 (57.8)	< 0.001
Yes	1046 (39.4)	115 (15.3)	61 (14.1)	162 (42.2)	

### The predictors for COVID-19 vaccine hesitancy among participants

According with results from the multivariate anal**yses **(Table 4), age was significantly associated with hesitancy, with older age being more likely to have COVID-19 vaccine hesitancy (OR: 0.974, 95% CI: 0.956 -0.993). Gender was significantly associated with hesitancy, female was more likely to be hesitant about COVID-19 vaccine than male (OR: 1.331, 95% CI: 1.050–1.687). Among different population groups, there were no statistical differences in the comparison of the other three populations and the general population (all *P* > 0.05). Those who agreed that the vaccine would protect them from the disease were less hesitant to receive the COVID-19 vaccine (OR: 0.404, 95% CI: 0.301–0.543). Participants who ever hesitated or refused to get vaccination were more likely to be hesitant (*P* < 0.05). The results showed that respondents' concerns about the infection with COVID-19 were associated with hesitancy, and those with higher self-report scores were more likely to be hesitant (OR: 1.086, 95% CI: 1.032–1.143). Higher scores of the General Vaccine Hesitancy Scale were associated with greater hesitancy towards COVID-19 vaccination (OR: 1.043, 95% CI: 1.029–1.056). Participants who will get COVID-19 vaccine if doctors recommend were less likely to hesitate (OR: 0.176, 95% CI: 0.132–0.234). However, participants whose vaccination intentions would be influenced by recommendations from family and friends were more likely to be hesitant (OR: 1.533, 95% CI: 1.212–1.940). Those who thought that they have no need of getting COVID-19 vaccine because they are healthy were more likely to be hesitant (OR: 1.938, 95% CI: 1.495–2.512). Participants who had doubts about the source of information (OR: 2.517, 95% CI: 1.697–2.742) and received negative information about the vaccine before (OR: 1.563, 95% CI: 1.229–1.986) were more likely to have COVID-19 vaccine hesitancy. Participants who needed transparent information about COVID-19 vaccine and would get COVID-19 vaccine after receiving the negative information were both less likely to be hesitant to get COVID-19 vaccine (*P* < 0.05).

**Table 4:** Predictors of COVID-19 vaccine hesitancy among participants (n = 4227)

Variables	Univariate analysis			Multivariate analysis	
	No Hesitancy (n = 3566)	Hesitancy (n = 661)	*P*	OR^*^ (95%CI^*^)	*P*
Age (years), ($mean\pm SD)$	33.59 $\pm$ 10.04	29.92 $\pm$ 8.48	< 0.001	0.974 (0.956–0.993)	0.006
Gender, n (%)					
Male	1219 (34.2)	190 (28.7)	0.006	1.000	
Female	2347 (65.8)	471 (71.3)		1.331 (1.050–1.687)	0.018
Ethnicity, n (%)					
Han	3169 (88.9)	596 (90.2)	0.325	─	
Other	397 (11.1)	65 (9.8)			
Residence place, n (%)					
Urban	3085 (86.5)	535 (80.9)	< 0.001	1.000	
Rural	481 (13.5)	126 (19.1)		0.959 (0.710–1.295)	0.785
Marital status, n (%)					0.822
Single	1392 (39.0)	352 (53.3)	< 0.001	1.000	
Married	2102 (58.9)	302 (45.7)		1.039 (0.745–1.449)	0.820
Others	72 (2.1)	7 (1.0)		0.774 (0.285–2.107)	0.617
Education level, n (%)					
≤ High school	208 (5.8)	52 (7.9)	0.046	1.000	
College and above	3358 (94.2)	609 (92.1)		0.771 (0.503–1.181)	0.232
Population groups					0.779
General population	342 (9.6)	92 (13.9)	< 0.001	1.000	
Students	573 (16.1)	180 (27.2)		1.219 (0.834–1.781)	0.306
Medical workers	2307 (64.7)	349 (52.8)		1.140 (0.801–1.624)	0.466
Public health professionals	344 (9.6)	40 (6.1)		1.177 (0.704–1.966)	0.535
Household income (past 1 year), n (%)					0.344
> 350,000	116 (3.3)	34 (5.1)	0.001	1.000	
110,000–350,000	1230 (34.5)	198 (30.0)		0.621 (0.362–1.063)	0.082
50,000–100,000	1562 (43.8)	273 (41.3)		0.627 (0.370–1.065)	0.084
≤ 40,000	658 (18.5)	156 (23.6)		0. 675 (0.386–1.179)	0.167
Ever smoking, n (%)					
No	3156 (88.5)	578 (87.4)	0.436	─	
Yes	410 (11.5)	83 (12.6)			
Ever drinking, n (%)					
No	3027 (84.9)	549 (83.1)	0.231	─	
Yes	539 (15.1)	112 (16.9)			
COVID-19 test results, n (%)					0.581
Positive	22 (0.6)	17 (2.6)	< 0.001	1.000	
Negative	2669 (74.8)	429 (64.9)		0.631 (0.256–1.558)	0.318
Haven’t tested	875 (24.5)	215 (32.5)		0.617 (0.249–1.531)	0.298
Do you agree that vaccines can protect you from diseases?					
No	268 (7.5)	150 (22.7)	< 0.001	1.000	
Yes	3298 (92.5)	511 (77.3)		0.404 (0.301–0.543)	< 0.001
Do you agree that you will get all vaccines that National Immunization Program or government recommended?					
No	654 (18.3)	268 (40.5)	< 0.001	1.000	
Yes	2912 (81.7)	393 (59.5)		0.783 (0.619–0.991)	0.042
Have you ever hesitated to get vaccination?					
No	2551 (71.5)	281 (42.5)	< 0.001	1.000	
Yes	1015 (28.5)	380 (57.5)		1.656 (1.302–2.106)	< 0.001
Have you ever refused to get vaccination?					
No	3117 (87.49)	436 (66.0)	< 0.001	1.000	
Yes	449 (12.6)	225 (34.0)		1.340 (1.022–1.756)	0.034
Have you ever hesitated or refused to be get Pneumococcal Vaccine?					0.042
No	2516 (70.6)	312 (47.2)	< 0.001	1.000	
Ever hesitated	784 (22.0)	257 (38.9)		1.450 (1.083–1.941)	0.013
Ever refused	266 (7.5)	92 (13.9)		1.340 (0.862–2.083)	0.193
Have you ever hesitated or refused to get Influenza Vaccine?					0.805
No	2525 (70.8)	346 (52.3)	< 0.001	1.000	
Ever hesitated	781 (21.9)	222 (33.6)		0.939 (0.698–1.262)	0.674
Ever refused	260 (7.3)	93 (14.1)		0.870 (0.563–1.344)	0.531
The General Vaccine Hesitancy Scale scores					
$m\pm SD$	19.12 $\pm$ 7.91	25.46 $\pm$ 8.52	< 0.001	1.043 (1.029–1.056)	< 0.001
Concerns about infection with COVID-19					
$m\pm SD$	9.77 $\pm$ 2.05	10.10 $\pm$ 2.05	0.001	1.086 (1.032–1.143)	0.002
Do you agree that COVID-19 epidemic is a severe problem affecting the health of the community?					
No	962 (27.0)	217 (32.8)	0.002	1.000	
Yes	2604 (73.0)	444 (67.2)		0.946 (0.741–1.208)	0.656
Do you agree that COVID-19 will be a great threat to your health if you are infected?					
No	377 (10.6)	93 (14.1)	0.009	1.000	
Yes	3189 (89.4)	568 (85.9)		1.212 (0.856–1.714)	0.279
Do you agree that you will get COVID-19 vaccine if doctors recommend?					
No	171 (4.8)	241 (36.5)	< 0.001	1.000	
Yes	3395 (95.2)	420 (63.5)		0.176 (0.132–0.234)	< 0.001
Do you agree that the advice of your family members or friends will affect your intention of getting COVID-19 vaccine?					
No	2260 (63.4)	271 (41.0)	< 0.001	1.000	
Yes	1306 (36.6)	290 (59.0)		1.533 (1.212–1.940)	< 0.001
Do you agree that you have no need of getting COVID-19 vaccine because you are healthy?					
No	3209 (90.0)	438 (66.3)	< 0.001	1.000	
Yes	357 (10.0)	223 (33.7)		1.938 (1.495–2.512)	< 0.001
Do you need transparent information about COVID-19 vaccine development, efficacy and safety?					
No	557 (15.6)	141 (21.3)	< 0.001	1.000	
Yes	3009 (84.4)	520 (78.7)		0.722 (0.535–0.973)	0.032
Do you have doubts about the source of information about the COVID-19 vaccine?					
No	2788 (78.2)	297 (44.9)	< 0.001	1.000	
Yes	778 (21.8)	364 (55.1)		2.157(1.697–2.742)	< 0.001
Have you ever received negative information about getting the COVID-19 vaccine?					
No	1725 (48.4)	251 (38.0)	< 0.001	1.000	
Yes	1841 (51.6)	410 (62.0)		1.563 (1.229–1.986)	< 0.001
Would you like to get the COVID-19 vaccine after receiving the negative information about it?					
No	1131 (31.7)	467 (70.7)	< 0.001	1.000	
Yes	2435 (68.3)	194 (29.3)		0.315 (0.249–0.398)	< 0.001
Do you agree that the time costs in waiting for the vaccination or staying at the clinic will be a barrier for you to get COVID-19 vaccine?					
No	2658 (74.5)	390 (59.0)	< 0.001	1.000	
Yes	908 (25.)	271 (41.0)		1.137 (0.870–1.487)	0.348
Do you agree that the environment of the clinic will be a barrier for you to get COVID-19 vaccine?					
No	2166 (60.7)	298 (45.1)	< 0.001	1.000	
Yes	1400 (39.3)	363 (54.9)		1.286 (0.986–1.679)	0.064
Do you agree that the cost of going to the clinic will be a barrier for you to get COVID-19 vaccine?					
No	2561 (71.8)	367 (55.5)	< 0.001	1.000	
Yes	1005 (28.2)	294 (44.5)		1.097 (0.839–1.434)	0.498
Have you gotten emergency COVID-19 vaccination for some reasons?					
No	2343 (65.7)	500 (75.6)	< 0.001	1.000	
Yes	1223 (34.3)	161 (24.4)		0.807 (0.626–1.040)	0.097

## Discussion

In current study, 15.6% of all participants self-reported having COVID-19 vaccine hesitancy, the rate was higher than the rate previously investigated in China (8.7%), however, it was lower than the findings that 22.4% in France, 25.5% in India, 29.2% in Italy and 37.4% in Ethiopia [31–34]. Among the study participants, the students had the highest rate (23.9%) of hesitancy, followed by general population (21.2%), medical workers (13.1%), and public health professionals (10.4%). Apart from changing attitudes to the increasingly severe COVID-19 pandemic and different study time, different socio-demographic factors, levels of health literacy, particularly with respect to immunization schedules, changes in local health policies and advances in the development of the COVID-19 vaccines may account for the differences on COVID-19 vaccine hesitancy. At present, the COVID-19 vaccine is available in China and the effectiveness of COVID-19 vaccination had been proved, however, this level of hesitancy among the population might be a barrier to the establishment of herd immunity.

The current study demonstrated that there were no significant differences among different populations in terms of the level of vaccine hesitancy. All individuals may have concerns on safety and efficacy issues of COVID-19 vaccination, as the COVID-19 vaccines were not undergone long-term clinical trials as other vaccines, the concern is one source of vaccine hesitancy [35–37]. Apart from widespread worries about vaccine safety and effectiveness, previous immunization behaviors can also influence vaccine uptake. Participants who have ever hesitated or refused to get vaccination were more likely to be hesitant for the vaccination against COVID-19 in our study. Similarly, another survey found that whether a person had the flu shot was a prominent determinant of COVID-19 vaccine avoidance [38]. And our results showed that participants who scored higher on the General Vaccine Hesitancy Scale were more likely to have hesitancy about the COVID-19 vaccine. In conclusion, the causes of vaccine hesitancy are complex and various, interventions should be developed to address different populations' hesitancy, especially the common causes. Timely health education and communication conducted by authoritative sources with corresponding explanations about their side effects will be critical to alleviate public concerns about vaccine safety [39, 40]. In addition, targeted interventions for those who have previously hesitated to receive the vaccine to enhance their willingness to undergo vaccination, are also needed.

Recommendations from physicians functioned as a motivator for participants to get vaccinated, and people would get the COVID-19 vaccine if their physicians recommended it. Medical workers always serve as the most trusted advisors and influencers of vaccination decisions, studies also demonstrated that those who valued doctor’s recommendations tended to get vaccinated immediately [31, 41]. However, doctors may be reluctant to spend time discussing vaccination with their patients, or they may feel they do not have enough information to address the issues that arise when recommending vaccines to their patients [42]. And if doctors themselves are hesitant about vaccines, their willingness to recommend vaccines to others may be reduced [43–45]. Even worse, their apprehensive attitude toward vaccines may exacerbate patients’ worries and doubts about vaccination [46]. Hence, educational campaigns based on accurate, authoritative knowledge of vaccines, maintaining a trustworthy patient-provider relationship, and training in communication practices might be working for building their own confidence in vaccines and willingness to recommend vaccines to others [43, 47].

Moreover, our study highlighted the needs of transparent information about COVID-19 vaccine development, efficacy, and safety. During the COVID-19 pandemic, governments and the public were unavoidably exposed to huge amount of rapid and far-reaching spread of questionable information, namely infodemic. Some media or websites exaggerated or made unreliable news on vaccination to attract more followers and negative information will be the possible sources of the increase in mistrust about vaccines[48–50]. Participants who received negative information about getting the COVID-19 vaccine were more likely to be hesitant in our study, which is consistent with previous studies [51, 52]. As the role of social media in public health promotion has grown, public health departments can also utilize the Internet for authoritative information dissemination, which will produce a positive impact on public health crises and vaccine campaigns [53, 54]. Medical workers are historically and uniformly important drivers of vaccine uptake, the information from them is generally accepted as authoritative, so the messages they send using social media platforms will be the primary information that informs the public's decision to vaccinate [55]. And they are essential to facilitate the interconnectedness of the general public, medical and public health communities on COVID-19. Therefore, medical workers can provide correct and accurate information, clarify misinformation through Internet to improve public’s confidence in vaccination. What's more noteworthy is that medical workers should be trained in the use of social media and be extremely careful in disseminating their opinions because their opinions are generally considered reliable, misinformation from them will mislead the public into making negative decisions [55]. And establishing suitable information exchange platform between the government and the public would be helpful to create a trustworthy environment, thus increasing the uptake of vaccines.

To overcome vaccine misinformation on social media, public health departments need to accurately monitor the spread of misinformation about vaccination in social media, which will not only help explore people's major concerns but combat false beliefs about vaccination timely. And public health-related authorities should establish an online platform that generates strategic tweets to fill knowledge gaps and leverage authoritative experts or respected celebrities to promote the benefits of vaccines to increase public’s willingness of vaccination [56, 57]. More importantly, educating social media users on how to distinguish reliable information and to encourage them not to circulate false information are helpful for tackling COVID-19-related misinformation [58]. Efforts by the government and public health community to maintain correct and authoritative messaging throughout the vaccine development and administration phases will be an effective measure for us to prevent misinformation from spreading, reduce vaccine hesitancy, and increase vaccination rates.

## Limitations

Our survey was conducted before the COVID-19 vaccine became widely available in China. Therefore, differences between the prevalence of hesitancy in our study and the actual vaccination rate later on might exist. However, our study still provided meaningful results in terms of the prevalence and related factors of the vaccine hesitancy among various populations. Although four different population groups were surveyed in our study, the generalizability of our results will still be limited in certain aspects due to the nonprobability sampling we used. Also, the questionnaires were self-reported online, thus the information bias probably existed in this study.

## Conclusions

Given information from healthcare professionals about vaccination recommendations will have a positive impact on public health crises and vaccine campaigns, providing appropriate information to them and training on communicating skills are of most important for their own benefit and the public’s. Since the negative information of COVID-19 vaccine could cause and increase vaccine hesitancy, transparent and authoritative information about the vaccine was vital for public to make vaccination decisions. Evidence-based information strategies where the potential benefits and risks of vaccination are properly explained, reducing the spread of misinformation and disseminating facts in a timely and accurate way, will likely reduce vaccine hesitancy.

## Supplementary Information

**Additional file 1: Table S1.** Scale items description (the COVID-19 vaccine hesitancy scale).

## Data Availability

The datasets used and/or analysed during the current study are available from the corresponding author on reasonable request.

## Abbreviations

CDC: Centers for Disease Control and Prevention
CI: Confidence interval
COVID-19: Coronavirus disease 2019
OR: Odds ratio
VHS: Vaccine Hesitancy Scale
WHO: World Health Organization
