# Supplementary material for: COVID-19 vaccine hesitancy among different population groups in China: a national multicenter online survey
Source: BMC Infect Dis. 2022 Feb 14;22:153. doi: 10.1186/s12879-022-07111-0 (PMC8845236; doi:10.1186/s12879-022-07111-0)
Supplement: Supplementary file 1 — Additional file 1: Table S1. Scale items description (the COVID-19 vaccine hesitancy scale). [file 12879_2022_7111_MOESM1_ESM.docx]

**Additional file 1**

| **Table S1 Scale items description (the COVID-19 vaccine hesitancy scale)** | | | | |
| --- | --- | --- | --- | --- |
|  | **Scale item** | **Response** | | |
|  |  | Hesitant responses  (2 points) | Not sure or not know  (1 points) | Non-hesitant responses  (0 points) |
| T1 | Would you delay getting the COVID-19 vaccine for reasons other than illness or allergy? | Yes | Don’t know^*^ | No |
| T2 | Would you decide not to get the COVID-19 vaccine for reasons other than illness or allergy? | Yes | Don’t know^*^ | No |
| T3 | would you want to get the nationally recommended COVID-19 vaccine? | 0-5 ^a^ | 6-7 | 8-10 |
| T4 | It is not necessary to get the COVID-19 vaccine at this time? | Agree ^b^ | Not sure | Disagree |
| T5 | I believe that the COVID-19 vaccine can prevent COVID-19 (COVID-19 is a severe disease). | Disagree | Not sure | Agree |
| T6 | It is better for you to develop immunity by getting COVID-19 than to get the COVID-19 vaccine. | Agree | Not sure | Disagree |
| T7 | It is better for you to get fewer vaccines at the same time. | Agree | Not sure | Disagree |
| T8 | How concerned are you that you might have a serious side effect from getting the COVID-19 vaccine? | Concerned ^c^ | Not sure | Not Concerned |
| T9 | How concerned are you that the COVID-19 vaccine might not be safe? | Concerned | Not sure | Not Concerned |
| T10 | How concerned are you that a shot might not prevent the COVID-19? | Concerned | Not sure | Not Concerned |
| T11 | If the COVID-19 vaccine becomes available, would you recommend your friends and family members to get the COVID-19 vaccine? | No | Not sure | Yes |
| T12 | Overall, how hesitant about the COVID-19 vaccine would you consider yourself to be? | Hesitant ^d^ | Not sure | Not hesitant |
| T13 | I trust the information I receive about the COVID-19 vaccine | Disagree | Not sure | Agree |
| T14 | I am able to openly discuss my concerns about shots the COVID-19 vaccine with doctor. | Disagree | Not sure | Agree |
| T15 | All things considered, how much do you trust doctor? | 0-5 ^e^ | 6-7 | 8-10 |
| ^*^ ‘don’t know’ response was excluded as missing data.  ^a^ Response category on a 0–10 scale, with 0 being ‘not at all sure’ and 10 being ‘completely sure’.  ^b^ Agree reflects combined responses of strongly agree and agree; disagree reflects combined responses of strongly disagree and disagree.  ^c^ Concerned reflects combined responses of very and somewhat concerned; not concerned reflects combined responses of not concerned at all and not too concerned.  ^d^ Hesitant reflects combined responses of very and somewhat hesitant; not hesitant reflects combined responses of not hesitant at all and not too hesitant.  ^e^ Response category on a 0–10 scale, with 0 being ‘do not trust at all’ and 10 being ‘completely trust’. | | | | |
